# Herpes Simplex Virus Type 1 (HSV-1) UL38 Capsid Protein: A Promising Target Against Viral Infection

**DOI:** 10.3390/v18060596

**Published:** 2026-05-25

**Authors:** Amalia A. Sofianidi, Fotios G. Spiliopoulos, Kostas A. Papavassiliou, Athanasios G. Papavassiliou

**Affiliations:** 1 Department of Biological Chemistry, Medical School, National and Kapodistrian University of Athens, 11527 Athens, Greece; amaliasof@med.uoa.gr (A.A.S.); spiliopoulosfotis99@gmail.com (F.G.S.); 2 First University Department of Respiratory Medicine, ‘Sotiria’ Chest Hospital, Medical School, National and Kapodistrian University of Athens, 11527 Athens, Greece

Herpes simplex virus type 1 (HSV-1) is a neurotropic virus that establishes lifelong latent infection. After primary infection, HSV-1 genomes persist in a latent state, particularly within neurons of sensory and autonomic ganglia, while retaining the capacity for reactivation [1]. According to the World Health Organization (WHO), approximately 3.8 billion people under 50 years of age, corresponding to 64% of the global population, are infected with HSV-1 [2]. Clinical manifestations range from recurrent oral or genital lesions [3] to severe disease, including keratitis [4] and encephalitis [5]. Accumulating evidence also supports an association between HSV-1 infection and neurodegenerative disorders, including Alzheimer’s disease [6], although causality and underlying mechanisms remain under active investigation. Current antiviral options, mainly nucleoside analogs, inhibit viral DNA polymerase but do not eradicate latent viral reservoirs. In addition, their clinical use can be limited by drug resistance, toxicity, and incomplete suppression of viral shedding [7]. Therefore, therapeutic strategies targeting additional steps in the HSV-1 life cycle, including viral immune-evasion mechanisms, are needed to address existing treatment gaps and reduce the public health burden of HSV-1 infection.

Herpesviruses package their double-stranded DNA genomes within an icosahedral nucleocapsid assembled in the host–cell nucleus. The HSV-1 capsid consists of the major capsid protein VP5, the triplex proteins VP19C and VP23, the small capsid protein VP26, and the scaffolding proteins VP21, VP22a, and VP24 [8]. After capsid assembly and genome packaging, the virus exits the nucleus and undergoes further maturation at secondary sites, acquiring tegument proteins and a lipid envelope containing viral glycoproteins [9]. VP19C, also known as unique long region 38 (UL38), is a critical component of capsid assembly and productive replication [10]. UL38 is a 465-amino-acid protein [10] that has recently attracted attention because of its direct interaction with the cyclic GMP-AMP synthase (cGAS)-stimulator of interferon genes (STING) pathway, through which it interferes with host innate immune signaling [11]. As a central DNA-sensing pathway of the innate immune system, cGAS-STING responds to infection, cellular damage, and tumorigenesis. Upon activation, cGAS catalyzes the production of cyclic GMP-AMP (cGAMP), which potentiates STING, recruits TANK-binding serine/threonine kinase 1 (TBK1), and induces type I interferon and pro-inflammatory cytokine production, as well as autophagy or apoptosis under specific cellular contexts [12]. HSV-1 UL38 acts as a STING antagonist by disrupting the interaction between STING and downstream signaling partners, thereby suppressing host antiviral responses [11].

Pharmacological targeting of viral cGAS-STING evasion mechanisms represents an attractive antiviral strategy [13]. Recent work has described therapeutic peptides designed to interfere with the interface through which UL38 binds STING [11,14]. In particular, the L4P peptide targets the Leu-Ile-Leu-Pro (LILP) motif within the STING 161–190 region and disrupts the UL38−STING interaction. This restores STING signaling and promotes the transcriptional activation of immune-related genes. The antiviral activity of L4P has been observed in human cell lines and in murine models of HSV-1 infection, where treatment increased survival and attenuated body-weight loss [11]. Antiviral effects were also reported in a mouse model of cerebral HSV-1 infection. In that setting, pathological evaluation of infected brains showed an increased accumulation of cluster of differentiation 11b+ (CD11b+) inflammatory cells [11]. This finding should be interpreted cautiously because recruitment of inflammatory cells may reflect enhanced antiviral immunity but may also contribute to immunopathology, depending on timing, localization, and magnitude. Nevertheless, such observations are relevant given that, although acyclovir (a synthetic purine nucleoside analog antiviral derived from guanine) has reduced HSV encephalitis-related mortality and morbidity, survivors may still experience severe neurological sequelae [15], and acyclovir-associated neurotoxicity remains a clinical concern, particularly in vulnerable patients [16].

A potential concern associated with therapeutic restoration of STING signaling is excessive or prolonged immune activation. Available preclinical evidence suggests that L4P enhances immune-related transcription early during HSV-1 infection but that this immunostimulatory effect gradually diminishes [11]. This is consistent with the proposed mechanism of action: L4P does not function as a direct STING agonist [17] but rather relieves UL38-mediated suppression of cGAS-STING signaling. As viral genome production declines, the degree of L4P-mediated STING activation also appears to decrease, suggesting a negative-feedback relationship between antiviral efficacy and immune stimulation [11]. Similar observations in murine models of cerebral infection support the possibility that UL38 targeting may restore antiviral signaling without causing sustained inflammatory activation in the brain [11]. Nonetheless, additional studies are required to define the therapeutic window, dose–response relationships, tissue-specific inflammatory consequences, and safety profile of this approach.

Acyclovir and related nucleoside analogs remain the standard of care for HSV-1 infection. However, because HSV-1 establishes lifelong infection, repeated or prolonged antiviral exposure can select for resistant variants, particularly in immunocompromised individuals. Consistent with acyclovir’s mechanism of action, resistance-associated mutations have been identified mainly in the *UL23* gene, which encodes viral thymidine kinase, and in *UL30*, which encodes the viral DNA polymerase [18]. Targeting the UL38-STING interface could, in principle, exert a different selection pressure from polymerase inhibitors. If mutations in UL38 disrupt peptide binding while simultaneously compromising STING antagonism or capsid assembly, viral fitness may be reduced. However, resistance cannot be excluded. HSV-1 could potentially evolve compensatory mutations, alter interaction surfaces without fully losing UL38 function, or rely on additional immune-evasion mechanisms. Therefore, resistance studies, serial-passage experiments, and mapping of escape mutants will be essential before this strategy can be considered clinically robust.

UL38 is classified as a true late (gamma-2) gene and is expressed during the late phase of lytic replication [10]. By the time late gene expression occurs, viral DNA replication has already generated multiple genome copies, and infection may have progressed toward viral spread and latency establishment. Late genes are transcriptionally silent during latency but can be re-expressed during reactivation [10]. Consequently, UL38 targeting is unlikely to prevent the initial establishment of latency. Its more plausible therapeutic role would be to reduce viral propagation, tissue damage, and disease severity during active infection or reactivation episodes. Given the limitations of suppressive nucleoside-analog therapy, including continued viral shedding during treatment and loss of protection after discontinuation [19], approaches that target late-stage replication or immune evasion may have value as adjunctive therapies. In this context, UL38-directed agents could potentially be used alone or, more realistically, in combination with polymerase inhibitors to target complementary stages of the viral life cycle. This concept is broadly analogous to combination antiviral strategies used in other chronic viral infections, although HSV-1 biology, latency, and treatment goals differ substantially from those of retroviral infections [20].

Previous attempts to therapeutically target UL38 have also been reported. Silencing HSV-1 capsid-protein genes with small interfering RNAs (siRNAs), including UL38, demonstrated antiviral potential but did not fully halt viral DNA replication [21]. Direct targeting of UL38 protein interactions, therefore, offers a mechanistically distinct approach. Peptide-based therapeutics directed against UL38 are promising, but several translational barriers must be addressed. UL38 is an intracellular protein, and poor membrane permeability remains a major limitation of many peptide therapeutics [22]. Additional challenges include peptide stability, pharmacokinetics, intracellular delivery, tissue distribution, blood–brain barrier penetration in neurological disease, immunogenicity, and scalable formulation. Another important consideration is viral specificity. Acyclovir is active against both HSV-1 and HSV-2, which is clinically convenient because the two viruses can cause overlapping mucocutaneous disease. By contrast, the STING-interacting region of HSV-2 UL38 differs from that of HSV-1 UL38 by one amino acid within the amino acid 81–90 region, implying that L4P may be less broadly applicable to HSV-2 [11]. Future optimization should therefore aim to improve intracellular delivery, define cross-reactivity against HSV-2, and determine whether wider-spectrum formulations can target related alphaherpesvirus immune-evasion mechanisms.

In conclusion, HSV-1 remains a major global health burden because it establishes lifelong infection and can cause recurrent or severe disease. Although nucleoside analogs remain central to HSV-1 therapy, they do not eliminate latent infection and may be limited by incomplete suppression of viral shedding, toxicity, and resistance. UL38, a capsid-associated HSV-1 protein involved in viral assembly and STING antagonism, has recently emerged as a druggable target. Preclinical peptide-based strategies that disrupt the UL38-STING interaction restore antiviral innate immune signaling and suppress HSV-1 pathogenesis in experimental models. These findings support UL38 as a promising target for antiviral development. However, further work is required to validate efficacy across clinically relevant models, establish safety and delivery feasibility, evaluate resistance potential, and determine whether UL38-targeted agents can complement existing polymerase inhibitors in future HSV-1 treatment strategies.

## Data Availability

Not applicable.
